# Schistosomiasis and urinary bladder cancer in North Western Tanzania: a retrospective review of 185 patients

**DOI:** 10.1186/1750-9378-8-19

**Published:** 2013-05-24

**Authors:** Peter Fabian Rambau, Philipo L Chalya, Kahima Jackson

**Affiliations:** 1 Department of Pathology, Catholic University of Health and Allied Sciences-Bugando (CUHAS-Bugando), Box 1464, Mwanza, Tanzania; 2 Department of Surgery, Bugando Medical Center, Mwanza, Tanzania

**Keywords:** Schistosomiasis, Urinary bladder cancer, Northwestern Tanzania

## Abstract

**Introduction:**

Worldwide, cancers of the urinary bladder are well known to be associated with environmental chemical carcinogens such as smoking and occupational exposure to polycyclic aromatic hydrocarbons. These cancers are typically transitional cell carcinoma (urothelial carcinoma). In areas where schistosomiasis is endemic there is a high incidence of squamous cell carcinoma of the urinary bladder. Schistosomiasis causes chronic granulomatous cystitis leading to squamous metaplasia of transitional epithelium, and subsequently development of squamous cell carcinoma. The western part of Tanzania on the shores of Lake Victoria is such an endemic area. This study was done to document the burden of urinary bladder cancer associated with schistosomiasis in this region.

**Methods:**

This was a descriptive retrospective study of histologically confirmed cases of urinary bladder cancer seen at the Department of Pathology Bugando Medical Centre (BMC) over a period of 10 years. Data were retrieved from the records of the Departments of Pathology, Medical Records and Surgery. Data were analyzed by the use of contingency tables.

**Results:**

A total of 185 patients were diagnosed with cancer of the urinary bladder during the study period, where as 90 (48.6%) were males and 95 (51.4) were females. The mean age at diagnosis was 54.3 years. Squamous cell carcinoma was the most frequent histological type (55.1%), followed by conventional transitional cell carcinoma (40.5%). Eighty three of all cancer cases (44.9%) were found to have schistosomal eggs. Schistosomiasis was commonly associated with squamous cancers compared to non squamous cancers. Most of the cancers associated with schistosomiasis had invaded the muscularis propria of the urinary bladder at the time of diagnosis (p<0.001) and such cancers were frequent below 50 years of age with a significant statistical difference (p<0.001). Poorly differentiated tumors were more frequent in females than males with a significant statistical difference (p=0.006).

**Conclusion:**

The majority of urinary bladder cancers seen in the Lake Region were squamous cell carcinoma associated with schistosomiasis. These cancers showed an aggressive behavior and were commonly seen in the younger age groups. Effective control of schistosomiasis in this region should significantly reduce the burden of urinary bladder cancer.

## Background

Schistosomiasis causes significant morbidity and mortality worldwide. It is estimated that about 600 million peoples living in the tropics are at risk of infection and 200 million are infected [1]. *Schistosoma haematobium*, which is the agent for urinary schistosomiasis, is commonly found in Sub-Saharan Africa and the Middle East. The peak prevalence and intensity of early infection occurs between the ages of 10 and 20 years and declines by the age of 65 years [2]. Adult *S. hematobium* commonly invade the venous plexus around the urinary bladder, the eggs released by adult worms cause chronic granulomatous inflammation in the mucosal and submucosal of the urinary bladder. Chronic granulomatous inflammation and irritation subsequently lead to the development of squamous metaplasia of the transitional epithelium. Chronic granulomatous inflammation also leads to bladder fibrosis which causes urine stasis and bacteria super infection, bacterials convert dietary nitrates and nitrites into nitrosamines which are then excreted in the urine. These nitrosamines are carcinogenic and act the on metaplastic epithelium, with a subsequent progression to squamous cell carcinoma [3]. The infection can spread and involve the ureters and kidneys causing chronic obstructive disorders and renal failure [4].

Squamous cell carcinoma (SCC) of the urinary bladder is strongly associated with schistosomal infection. In schistosomal endemic areas of Africa, the prevalence of SCC is high compared to conventional transitional cell carcinoma (TCC), especially in rural areas [5,6]. In Egypt, cancers of the urinary bladder accounts for 30.3% of all cancers, of which the majority are squamous cell carcinoma related to schistosomal infection. This is similar to other African countries, such as Sudan, Kenya, Uganda, Nigeria and Senegal [7-9]. Although there are many areas in Tanzania where *S. hematobium* is endemic, the prevalence of squamous cell carcinoma of the urinary bladder in North-Western Tanzania has not been previously documented. In the Northern part of Tanzania, the distribution of *S. hematobium* is patchy, with areas of high and low endemicity. One study showed that the proportion of SCC was 72% in high endemic areas, with 46% of the cases positive for schistosomes eggs. In areas of low endemicity around Mount Kilimanjaro, SCC rarely occurs [10]. In areas where the control of *S. hematobium* has been effective there is a decrease in incidence of bladder tumors, with changing of histological profile from squamous cell carcinoma to transitional cell carcinoma [11].

Schistosoma-associated urinary bladder cancer with positive schistosomal eggs tends to occur at a relatively young age with a high tendency towards bladder muscle invasion, compared to non schistosomal associated cancers in western countries [12-14]. A recent study in Egypt showed a changing trends of the urinary bladder tumor incidence as a result the control of schistosomal infection. The trends showed an increased median age of diagnosis from 47.4 years to 60.5 years, with a decrease in SCC. TCC was the commonest form of cancer with low eggs positivity [11]. Other studies also showed the tendency for a decrease in SCC and trends towards TCC following effective control of schistosomiasis, and patients were much older [15-17]. A study in Nigeria showed the SCC was the most common form of bladder cancer, with a median age at diagnosis of 46 years of age. The majority of the patients were farmers and fishermen living in regions along the river, whereby 50% of the cases were positive for schistosomes eggs [9]. In general, *S. hematobium* has been related, epidemiologically and clinically to bladder carcinoma, specifically SCC. This study aimed at determining significance of the association between bladder cancer and schistosomiasis in a region where there is a high prevalence of urinary schistosomiasis.

## Methods

This was a descriptive, retrospective study of histologically confirmed cases of urinary bladder cancer, seen at the Department of Pathology of Bugando Medical Centre (BMC) over a period of 10-years between January 2000 and September 2010. BMC is a consultant, tertiary care and teaching hospital for the Catholic University of Health and Allied Sciences-Bugando (CUHAS-Bugando) with a bed capacity of 900. Subjects of this study included all histologically confirmed cancers of urinary bladder made during the study period. Data was retrieved from the records of the Pathology Department. Histology slides were re-evaluated for the type, grade and presence of schistosoma eggs. Patients’ files kept in the Medical Records and Surgical department were also used for clinical and demographic information. Patients with incomplete data (complete clinical history, age, failure to retrieve histology slides) were excluded from the study. Ethical approval to conduct the study was obtained from the CUHAS-Bugando/BMC joint institutional ethic review committee before the commencement of the study. Data were entered and analyzed using SPSS computer software version 15 and STATA 11. Student *t*-test and Chi square tests were used to establish associations, and significant association was considered when p value was less than 0.05.

## Results

During the study period, 185 patients were diagnosed with cancer of the urinary bladder, 90 (48.6%) men and 95 (51.4) women. The age ranged from 23 years to 98 years with mean age at diagnosis being 54.3 years, 63 (34.1%) of the patients were over 61 years; and 77(41.6%) of the patients were below 50 years of age. The mean age for the diagnosis of bladder cancer in males was 56.6 years while in females it was 52.2 years with no significant difference (p=0.055). Forty eight (48) females seen in the study were below 50 years of age compared to the males who were only 29, with a significant difference (See Table 1). The leading histological type of bladder cancer was the squamous cell carcinoma (SCC) 102 (55.1%), whereby 47 cases (46.1%) were found in males and 55 (53.9%) in females. Conventional urothelial carcinoma, i.e. TCC, was the second leading histological type which accounted for 75 cases (40.5%), and among them 37 (49.3%) were found in males and 38 (50.6%) in females (See Table 1). There was no significant difference in the distribution of the different types of cancers between males and females (See Table 1).

**Table 1:** Patients Gender in relation to demographic characteristics and pathological features

	**Sex**		
	**Male**	**Female**	**P- value**
	**N (%)**	**N (%)**	
**Age group**			
Below 50 years	29 (37.6)	48 ( 62.3)	0.017
Above 50 years	61 (56.8)	47 (43.5)	
**Histological type**			
Transitional cell carcinoma	37 (49.3)	38 (50.6)	
Squamous cell carcinoma	47 (46.1)	55 (53.9)	
Adenocarcinoma	4 (80.0)	1 (20.0)	0.450
Others	2 (66.6)	1 (33.3)	
**Presence of schistosomal eggs**			
Yes	34 (40.9)	49 (59.0)	0.076
No	56 (54.9)	46 (45.0)	
**Muscle invasion**			
Yes	55 (44.4)	69 (55.6)	0.118
No	35 (57.3)	26 (42.6)	
**Tumor differentiation**			
Well differentiated	30 (65.2)	16 (34.8)	
Moderately differentiated	33 (51.6)	31 (48.4)	0.006
Poorly differentiated	27 (36.0)	48 (64.0)	

When all other types of cancers were grouped together, non squamous cancers constituted only 44.9% of the cases. In all cancer cases, 83 (44.9%) were found to have schistosomes eggs. There was no sign of an active inflammation and most of the eggs were calcified, indicating old infections. Cases of squamous cell carcinoma (73.5%) were commonly associated with Schistosomes eggs compared to other types of cancers (See Table 2). The presence of schistosomes eggs did not differ between males and females (p=0.076), however, for both sexes, the mean age for presence of schistosomiasis was lower compared to patients with no schistosomiasis. In males, the mean age for presence of schistosomiasis was 49.6 years (95% CI, 44.40–54.77 p<0.001) while in females it was 45.2 years (95% CI, 41.36–49.08 p<0.001). For patients with no schistosomes eggs, the mean age was 60.8 and 59.7 years in males and females respectively (See Table 3). At the time of diagnosis, 124 (67%) of the tumors had already invaded the muscularis propria of the bladder, with no significant difference between males and females (p=0.118).

**Table 2:** Histological types of bladder cancer and presence of schistosomal eggs

	**Squamous cell carcinoma**	**Transitional cell carcinoma**	**Adenocarcinoma**	**Others**	**p-value**
**Presence of schistosomal eggs**	**N (%)**	**N (%)**	**N (%)**	**N (%)**	
Yes	75 (73.5)	7 (9.3)	1 (20)	0 (0)	< 0.001 **
No	27 (26.4)	68 (90.6)	4 (80)	3 (100)	
**Total**	102	75	5	3	

** (soma cells has count less than 5 this association can not be validated).

**Table 3:** Mean age of patients in relation to sex, type of cancer and presence of schistosomal eggs

		**95% CI**	**P -value**
**Sex**	**No (Mean age)**		
**Male**			
Schistosomal eggs present	34 (49.6)	44.40–54.77	
No schistosomal eggs	56 (60.8)	32.59–64.32	< 0.001
**Female**			
Schistosomal eggs present	49 (45.2)	41.36–49.08	
No schistosomal eggs	46 (59.7)	55.25–64.18	< 0.001
**Male**	**No (Mean age)**		
Non squamous cancer	43 (59.8)	56.45–63.13	
Squamous cancer	47 (53.6)	48.60–58.67	0.0473
**Female**			
Non squamous cancer	40 (62.4)	58.45–67.35	
Squamous cancer	55 (44.2)	41.09–47.82	< 0.001

Poorly differentiated tumors constituted 40.5% of all tumors, followed by moderately differentiated tumors (36.6%), and lastly by well differentiated tumors (24.8%). Well differentiated tumors tended to be more prevalent in males, while poorly differentiated tumors were more prevalent in females (p=0.006), (See Table 1). Schistosomiasis was more frequently found in squamous cancers cases compared to cases of non squamous cancers. The mean age for diagnosis of squamous and non squamous cancers did not differ between males and females. However, in both sexes, squamous cancers were seen in lower mean age compared to non squamous cancers and the difference was significant (See Table 3). When squamous cancers were compared with non squamous cancers, squamous cancers occurred more frequently in the age group below 50 (OR 0.12, 95% CI 0.06–0.28 p < 0.001) and were commonly associated with the presence of schistosomes eggs (OR 0.03, 95% CI 0.01–0.11 p< 0.001). Furthermore, squamous cancers showed a high tendency to invade the bladder wall muscles (OR 0.05, 95% CI 0.23–0.88 p=0.0167) compared to non squamous cancers. Moreover, squamous cancers appeared to be well differentiated compared to non squamous cancers (See Table 4).

**Table 4:** Types of cancers in relation to demographic characteristics and pathological features

	**Type of cancer**		**OR**	**(95% CI)**	**P -value**
	**Squamous**	**Non squamous**			
**Age group**	**N (%)**	**N (%)**			
Below 50 years	63 (81.8)	14 (18.1)	1.00		
Above 50 years	69 (63.8)	39 (36.1)	0.12	0.06–0.28	<0.001
**Presence of Schistosoma eggs**					
Yes	75 (90.3)	8 (9.6)	1.00		
No	27 (26.4)	75 (73.5)	0.03	0.01–0.11	<0.001
**Muscle invasion**					
Yes	76 (61.2)	48 (38.7)	1.00		
No	26 (42.6)	35 (57.3)	0.05	0.23–0.88	0.0167
**Tumor differentiation**					
Well differentiated	34 (73.9)	12 (26.0)	1.00		
Moderately differentiated	37 (57.8)	27 (42.1)	0.48	0.21–1.11	<0.001
Poorly differentiated	31 (41.3)	44 (58.6)	0.24	0.11–0.58	

## Discussion

This study found that the mean age at diagnosis for urinary bladder cancer, regardless of histological type, was 54.3 years of age. The mean age at diagnosis did not differ in both sexes. In Senegal, urinary bladder cancer is considered as a disease of young age, with a mean age at diagnosis being 45.5 years [18]. This study found that 41.6% of the patients were below 50 years of age. It is known that most of the bladder cancers present above 50 years of age, however this study found a trend towards a younger age groups at diagnosis for patients with bladder cancer.

Urinary bladder cancer is generally reported to be more common in males than in females. In this study, however, more females were affected (51.4%) compared to the males; this finding may be explained by different etiologies in specific settings. Bladder cancer is six times more common in developed countries than developing countries, and the majority (90% to 95%) are urothelial/transitional cell carcinoma [19,20]. In the current study, squamous cell carcinomas were more common (55.1%) than urothelial carcinoma, reflecting a different etiology in this settings compared to developed countries. Urothelial carcinoma has been associated with chemicals such as aniline dyes and aromatic amines associated with occupational exposure, a fact which is observed more frequently in developed countries than developing countries [21]. Other risk factors have been described, such as drugs (cyclophosphamide, phenacetin) and arsenic. In the present study, urothelial carcinoma constituted 48.6% of all bladder tumors. This suggests that chemical environmental exposure to the carcinogens implicated in urothelial cancers may not be as common as in developed countries. In the settings of this study, the chronic use of drugs, such as phenacetin, is difficult to ascertain but the use of cyclophosphamide is relatively low. Squamous cell carcinoma of the bladder has also being linked with other causes than schistosomiasis, such as chronic cystitis associated with urinary bladder stones, chronic indwelling catheters, and patients with spinal cord injury. It has also been seen in patients treated with cyclophospahamide. These particular types of carcinoma tend to occur in older age groups and be aggressive [22,23]. The finding of a relatively young age for patients with squamous cell carcinoma in this study suggests that these factors are not important in causation of squamous cell carcinoma in the Lake Zone area. This is also supported by the findings that schistosomal eggs and squamous cancers were common in lower mean age groups compared to urothelial and other cancers.

Squamous cell carcinoma, whether related to schistosomiasis or not, has been reported to be of distinct clinico-pathological features, with a high tendency of bladder wall muscle invasion, and advanced stages with lower incidence of pelvic node and distance metastasis than urothelial carcinoma [24].

Chronic cystitis associated with *Schistosoma haematobium* has been linked to squamous cell carcinoma of urinary bladder in many studies. Squamous cell carcinoma is more common in areas with a high prevalence of schistosomiasis compared to areas of low prevalence. This is commonly encountered in developing countries in areas with a high prevalence of schistosomiasis. In the present study, there were more cases of squamous cell carcinoma (51.4%) compared to urothelial carcinoma. Our study area is surrounded by the Lake Victoria, where schistosomiasis is highly prevalent. Squamous cell carcinoma was significantly more common in the age group below 50 years (p<0.001). This suggests that schistosomiasis plays a significant role in causation of urinary bladder cancer in this area. In Egypt, which is an industrialized country, urothelial carcinomas are commonly seen in urban areas. The control of schistosomiasis in the rural populations in Egypt led to a drop in the incidence of squamous cell carcinoma, confirming the role of schistosomiasis as a possible etiology in this type of cancer; and urothelial carcinoma showed a pattern similar to that of western countries [11,25]. This indicates that the control of schistosomiasis in similar settings has the potential to significantly reduce the burden of bladder cancer.

Further evidence from this study shows that among all bladder cancer detected, 44.9% had schistosomes eggs. The eggs were calcified with fibrosis around the eggs, indicating an old infection; furthermore, such eggs were more frequently found in squamous cancers compared to non-squamous cancers with a significant difference (p<0.001). This demonstrates that schistostosomiasis may be responsible for a large burden of urinary bladder cancers in the study area. In this study, adenocarcinoma constitutes only 2.7% of all cancers, and this does not differ from what is reported in the literature [26].

This study also observed that 67% of all the cancers had already invaded the bladder wall muscles at the time of diagnosis, thus reflecting an advanced stage with poor prognosis. The bladder muscle wall invasion was significantly associated with poorly differentiated tumors (high grade tumors), regardless of the histological types (p<0.001). This possibly reflects a delay in seeking medical care which is common in these settings. Furthermore, squamous cancers showed a higher tendency of bladder muscle invasion at diagnosis compared to non squamous cancers (p=0.00167), with no significant statistical difference between males and females. This might infer that squamous cancers are inherently more aggressive cancers than non-squamous cancers. A study undertaken in upper Egypt showed that muscle invasive bladder cancer was more common in squamous cancers than urothelial cancers [14]. The reason why bladder squamous cancers seem more aggressive than urothelial cancers has not yet been confirmed. This type of cancer has been regarded as aggressive carcinoma *de novo*[27], with some studies showing that squamous cell carcinoma presents with an advanced stage with over-expression of p53 and EGFR [28].

Findings of the current study concur with other studies undertaken to study the causal relationship between schistosomiasis and bladder cancer. In developing countries, were schistosomiasis is prevalent, the types of bladder cancer encountered, particularly in rural areas, are predominantly squamous cell carcinoma which are highly aggressive. They occur at a relatively young age, unlike in developed countries where the majority of the cancers are urothelial carcinoma associated with industrialization [6,18]. Studies have shown that an effective control of schistosomiasis, in settings where bladder squamous cancers are common, leads to changes of the histological types of cancers towards a western type [29]. In addition, the results of this study showed that squamous cell cancers were more common in rural districts, in particular the districts of Bariadi with 20 cases (19.6%) followed by Misungwi with 15 cases (14.7%) and Sengerema with 14 cases (13.7%).

## Conclusion

This study showed that the trend of urinary bladder cancer in this region does not differ from other regions where schistosomiasis is prevalent. The majority of the bladder cancers detected in this region were squamous cell carcinoma with aggressive behavior, commonly seen in young age groups. The squamous cancers were significantly associated with a schistosomiasis. An effective control of schistosomiasis in the region would therefore significantly reduce the burden of bladder cancer in this region.

### Ethical approval

The research proposal was presented to joint Bugando Hospital and CUHAS-Bugando ethic committee, research and publication where it was approved.

## Consent

This research was retrospective study, histological slides, tissue blocks and patients files were used to get the information, confidentiality of the patients was maintained, only histology numbers and file numbers were used, this was approved by joint Bugando Hospital and CUHAS-Bugando ethic committee.

## Competing interests

All authors declare that they have no competing interests.

## Authors’ contributions

PFR Main author of the study, involved in design, writing the proposal, data collection, pathological review, analysis and preparation of the manuscript. PLC Involved in preparation of the study, clinical data collection, and preparation of the manuscript. KJ Involved in development of proposal, together with main author in review of histological slides and manuscript preparation. All authors read and approved the final manuscript.
